# Second Language Speech Fluency: What Is in the Picture and What Is Missing

**DOI:** 10.3389/fpsyg.2022.859213

**Published:** 2022-02-28

**Authors:** Ruiling Feng, Qian Guo

**Affiliations:** ^1^Public English Teaching Department, Foreign Language College, Tianjin Normal University, Tianjin, China; ^2^Department of Foreign Languages and Literatures, Tsinghua University, Beijing, China

**Keywords:** L2 speech fluency, multidisciplinary, cognitive fluency, utterance fluency, perceived fluency, fluency in L2 dialogues

## Introduction

Fluent speech is essential for smooth communication, whereas second language (L2) speech fluency is rarely comparable to first language (L1) speech fluency (Segalowitz, 2010). The gap is caused by various factors and may lead to communication anxiety or even communication breakdown. Therefore, fluent speech is one of the ultimate goals of L2 teaching and learning. L2 speech fluency represents an essential aspect of L2 testing and research. One of the latest monographs on L2 speech fluency, *Second Language Speech Fluency: From Research to Practice* written by Tavakoli and Wright (2020), explores this topic from two perspectives, i.e., fluency as speech performance and fluency in interaction. The authors claim two aims of this book. First, this book aims to introduce definitions, theoretical frameworks, methodological principles, and relevant empirical studies of L2 speech fluency. The other aim is to promote a multidisciplinary perspective to connect research of fluency as a psychological concept with that of fluency as a social concept. The major contributions of this erudite yet reader-friendly book lie in reconceptualizing and systemizing L2 speech fluency and identifying research gaps with an updated systematic review of both research and practice. However, some important topics in this field do not find a place in this book. Below we review the contents and strengths of the book and discuss some missing topics that have attracted increasing scholarly attention but still warrant further research.

## Strengths of the Book

The book consists of eight chapters. Chapter 1 lays a foundation for the whole book by introducing conceptualizations and theoretical models. The authors reconceptualize fluency as a multi-component concept combining cognitive and social factors. They also identify two disconnects. One is the disconnect among different disciplines involved in L2 fluency research. The other is the disconnect between the research of L2 fluency and related practices. Chapter 2 elaborates on some widely-accepted fluency-related psycholinguistic paradigms and socio-cognitive factors. It introduces Levelt's speech production model (conceptualization, formulation, articulation, and monitoring), Segalowitz's triadic fluency model (cognitive fluency, utterance fluency, and perceived fluency), and Skehan's triadic utterance fluency framework (speed, breakdown, and repair). Fluency is also examined in broader psycho-social contexts. Chapter 3 recounts the operationalization and measurement of utterance fluency in terms of speed, breakdown, repair, and composite indices. Besides, it highlights the complex structures of dialogues and computer-assisted interactions, such as turn-taking, interlocutor factors, and speaker stance, which warrant further studies. Chapter 4 evaluates the effects of the immediate context on fluency from a task-based perspective. The authors discuss the effects of task design, implementation conditions, and interlocutor factors on L2 fluency. Chapter 5 focuses on fluency in L2 pedagogy, covering L2 policy documents, L2 textbooks, classroom practice, and teacher cognition. It synthesizes descriptions of fluency in second language benchmarks and curricula. This chapter also provides recommendations for classroom activities and points out the scarcity of research into teacher perception and understanding of fluency. Chapter 6 examines how fluency is measured in some international language tests in terms of rating scales and fluency descriptors. The authors discuss the gap between testing practices and fluency research and suggest assessing fluency with a more social and task-based approach. Then, this chapter reports the relationship between fluency and language proficiency. Chapter 7 explores L2 fluency in various language learning contexts and from a multilingual view by including non-English languages. The authors argue for a more realistic and authentic norm other than native speakers as the target in L2 learning. Chapter 8 recaps all the themes covered in previous chapters and suggests areas for future research. It reiterates the importance of a broad multidisciplinary perspective and the connection between research and practice.

The book brings together, in a single volume for the first time, an overarching review of L2 speech fluency. Six topics are included, namely psycholinguistic theories, operationalization and measurement, task-based approach, teaching practice, testing applications, and fluency development in different contexts. Except for the introduction and conclusion chapters, each of the other six chapters represents a high-quality meta-analysis review of one topic. The wide-range topics, multidisciplinary perspective, up-to-date synthesis, and accessible writing style render this book an encyclopedia-style manual with both theoretical implications and practical tools to various language-related stakeholders.

More importantly, this volume navigates future research opportunities which are tremendously inspirational. Three research gaps are worth special attention in future studies. Firstly, little is known about the operationalization and measurement of fluency in L2 dialogues, although dialogues are even more common than monologs in reality. This book suggests that researchers of dialogue fluency look beyond speaker-internal factors by including interlocutor factors, and beyond learner linguistic ability by evaluating their communicative ability. Secondly, another problem worth more attention is the disconnect between related disciplines in L2 fluency research. The authors advocate a broader socio-cognitive perspective on and multidisciplinary insights into research, teaching, and assessment of L2 fluency. The broader perspective makes this book the first one that discusses L2 fluency in terms of both cognitive factors such as automaticity and external factors such as task designs. Thirdly, fluency descriptors and scales in many high-stakes language tests predominantly focus on the rapidity, fluidity, and easiness of L2 speeches, while neglecting the interactional nature of fluency in dialogues. This may entail washback effects that L2 learners excessively focus on their speech production rather than engaging in interaction by responding to their interlocutors. Monologic fluency at the sacrifice of responsiveness between turns may cause communication breakdowns. Therefore, fluency should be assessed from two perspectives, i.e., fluency as speech performance and fluency in interaction, to enhance the validity and reliability of scoring in standardized speech tests.

### Missing Topics of L2 Speech Fluency

However, some promising topics in L2 fluency research are not included in this review-style monograph. First, this book does not specifically examine the operationalization and measurement of cognitive fluency and perceived fluency, two essential dimensions in Segalowitz's triadic fluency framework. These two dimensions could help understand the underlying production process, listeners' understanding of L2 fluency, and L2 fluency as a multidimensional concept. Research interest is emerging in recent years in cognitive fluency related to the four cognitive processes in Levelt's speech production model, L2-specific cognitive fluency in particular (e.g., Segalowitz, 2010, 2016; Kahng, 2014, 2020). L2-specific cognitive fluency is gained by partialling out the cognitive fluency in L1 from that in L2, given that L2 utterance fluency is affected by both L2 ability and language-independent personal speaking style (Segalowitz, 2016; Bradlow et al., 2017). Perceived fluency, especially that by multilingual raters against the native-speaking rater bias, has also attracted increasing scholarly attention (e.g., Rossiter, 2009; Magne et al., 2019).

Second, this book mentions fluency in L2 dialogues and the differences between this fluency mode and monologic fluency, but it does not synthesize common measures of fluency in L2 dialogues. McCarthy (2010) introduced the concept “confluence” to describe the co-constructed nature of a dialogue. The collaborative nature may be demonstrated by interruption, overlap, or long pauses, measured in terms of duration and frequency (Tavakoli, 2016; van Os et al., 2020). Peltonen (2017) proposed the concept of “dialogue fluency”. This concept is measured by the frequency and mean duration of turn pauses, the frequency of repetitions of the immediately previous speaker's utterance, and the frequency of collaborative completions (Peltonen, 2017; Foster, 2020). These measures mainly pertain to temporal characteristics and cannot properly reflect the interactional nature of dialogues. Tavakoli and Wright (2020) argued for a more social perspective to measure fluency in L2 dialogues, but they did not propose specific operationalization and measurement methods. We suggest combining the temporal measures of fluency as speech performance with the inter-turn responsiveness measure of fluency in interaction. Though what an interlocutor says typically should “link and provide continuity with the immediately previous talk” in a dialogue (McCarthy, 2010, p. 5), it might not be the case in language learning or language testing, especially in an L2 speech test environment. Including responsiveness in fluency assessment could encourage or push L2 speakers to be “other-oriented in paying attention to their interlocutor” and “take the initiative in constructing a meaningful effective dialogue” (Tavakoli and Wright, 2020, p. 37).

Third, this book does not review studies of the relationships between cognitive fluency, utterance fluency, and perceived fluency, but these studies contribute to a better understanding of L2 fluency as a multidimensional concept in both psycholinguistic and social domains. A prominent finding in research of this kind is that L2 utterance fluency relies on both L2-specific cognitive fluency and language-general personal speaking style measured as equivalent L1 utterance fluency (e.g., Sato, 2014; Kahng, 2017, 2020). Previous studies of perceived fluency often relate it to measures of utterance fluency and find a strong association between the two regardless of the language background of raters (de Jong et al., 2013), though results are inconclusive concerning what utterance fluency measures are associated with perceived fluency. Relating utterance fluency features to perceived fluency are important because the subjective judgment of an interlocutor's speech fluency might affect the willingness to communicate, and therefore it is essential to understand what features listeners attend to when determining perceived fluency (Segalowitz, 2016). Research gaps still exist in the exploration of relationships among the three dimensions. For example, to the best of our knowledge, the relationship between cognitive fluency and fluency in L2 dialogues has not been examined to date, nor has the relationship among all the three dimensions together.

## Conclusions

Despite the aforementioned imperfections, the book provides a comprehensive and cutting-edge review of the research and practice in L2 speech fluency. The research gaps identified by it could serve as a stepping-stone for further research. This volume is an invaluable manual-style reference for researchers, instructors, material writers, teacher educators, and test developers in language education, applied linguistics, and psycholinguistics. Meanwhile, besides the topics covered by the book, we also argue for more attention to the missing topics to advance research and practices related to L2 speech fluency.

## Author Contributions

RF drafted the manuscript. All authors revised the manuscript and have approved it for publication.

## Funding

This work was supported by the Project of Discipline Innovation and Advancement (PODIA)-Foreign Language Education Studies at Beijing Foreign Studies University (Grant Number: 2020SYLZDXM011), Beijing.

## Conflict of Interest

The authors declare that the research was conducted in the absence of any commercial or financial relationships that could be construed as a potential conflict of interest.

## Publisher's Note

All claims expressed in this article are solely those of the authors and do not necessarily represent those of their affiliated organizations, or those of the publisher, the editors and the reviewers. Any product that may be evaluated in this article, or claim that may be made by its manufacturer, is not guaranteed or endorsed by the publisher.

## References

[B1] Bradlow A. R. Kim M. Blasingame M. (2017). Language-independent talker-specificity in first-language and second-language speech production by bilingual talkers: L1 speaking rate predicts L2 speaking rate. J. Acoust. Soc. Am. 141, 886–899. 10.1121/1.4976044 28253679 PMC5848867

[B2] de Jong N. H. Steinel M. P. Florijn A. Schoonen R. Hulstijn J. N. (2013). Linguistic skills and speaking fluency in a second language. Appl. Psycholinguist. 34, 893–916. 10.1017/S0142716412000069

[B3] Foster P. (2020). Oral fluency in a second language: a research agenda for the next ten years. Lang. Teach. 53, 446–461. 10.1017/S026144482000018X

[B4] Kahng J. (2014). Exploring utterance and cognitive fluency of L1 and L2 English speakers: temporal measures and stimulated recall. Lang. Learn. 64, 809–854. 10.1111/lang.12084

[B5] Kahng J. (2017). The effect of pause location on perceived fluency. Appl. Psycholinguist. 39, 569–591. 10.1017/S0142716417000534

[B6] Kahng J. (2020). Explaining second language utterance fluency: contribution of cognitive fluency and first language utterance fluency. Appl. Psycholinguist. 41, 457–480. 10.1017/S0142716420000065

[B7] Magne V. Suzuki S. Suzukida Y. Ilkan M. Saito K. (2019). Exploring the dynamic nature of second language listeners' perceived fluency: a mixed-methods approach. TESOL Q. 53, 1139–1150. 10.1002/tesq.528

[B8] McCarthy M. (2010). Spoken fluency revisited. Eng. Prof. J. 1, 1–15. 10.1017/S2041536210000012

[B9] Peltonen P. (2017). Temporal fluency and problem-solving in interaction: an exploratory study of fluency resources in L2 dialogue. System 70, 1–13. 10.1016/j.system.2017.08.009

[B10] Rossiter M. J. (2009). Perceptions of L2 fluency by native and non-native speakers of English. Can. Mod. Lang. Rev. 65, 395–412. 10.3138/cmlr.65.3.39535182305

[B11] Sato M. (2014). Exploring the construct of interactional oral fluency: second language acquisition and language testing approaches. System 45, 79–91. 10.1016/j.system.2014.05.004

[B12] Segalowitz N. (2010). Cognitive Bases of Second Language Fluency. New York: Routledge. 10.4324/9780203851357

[B13] Segalowitz N. (2016). Second language fluency and its underlying cognitive and social determinants. IRAL-Int. Rev. Appl. Li. 54, 79–95. 10.1515/iral-2016-9991

[B14] Tavakoli P. (2016). Fluency in monologic and dialogic task performance: challenges in defining and measuring L2 fluency. IRAL-Int. Rev. Appl. Li. 54, 133–150. 10.1515/iral-2016-9994

[B15] Tavakoli P. Wright C. (2020). Second Language Speech Fluency: From Research to Practice. Cambridge University Press. 10.1017/9781108589109

[B16] van Os M. de Jong N. H. Bosker H. R. (2020). Fluency in dialogue: turn taking behavior shapes perceived fluency in native and nonnative speech. Lang. Learn. 70, 1183–1217. 10.1111/lang.12416

